# The interventions and outcomes associated with fall-related injuries at tertiary hospitals in the Kingdom of Saudi Arabia: a cross sectional study

**DOI:** 10.11604/pamj.2020.36.314.21943

**Published:** 2020-08-20

**Authors:** Sameer Al-Ghamdi, Ahmed Mohammed Alosaimi, Abdullah Omar Bin Shihah, Abdulrahman Ibrahim Alhadlaq, Musaad Abdullah Alotaibi, Ahmed Zaid Alnefaie, Faisal Musaad Alsaleh, Saad Fahad Alotaibi, Saud Fahad Alotaibi

**Affiliations:** 1 Department of Family Medicine, College of Medicine, Prince Sattam bin Abdulaziz University, Al-Kharj, Saudi Arabia,; 2 Shaqra College of Medicine, Shaqra University, Shaqra City, Saudi Arabia

**Keywords:** Accidental falls, wounds and injuries, case management, Saudi Arabia

## Abstract

**Introduction:**

fall-related injuries are an important health concern around the globe, imposing an immense economic burden. The aim of this study is to evaluate the interventions and outcomes associated with fall-related injuries in a tertiary hospital in the Kingdom of Saudi Arabia (KSA).

**Methods:**

a cross-sectional study including 264 patients with fall-related injuries was conducted at the King Khalid Hospital and Prince Sultan Centre for Health Care and other hospitals in Al Kharj from March 01, 2019 to November 30, 2019. The patients were recruited, identified at the point of presentation to the emergency department and followed through the triage, admission and discharge processes. The researchers analysed the participant´s clinical notes on the electronic health record (EHR) to obtain information relevant to the study, including demographic information, the injury patterns and their management.

**Results:**

most patients studied were children under the age of 10 (25.7%). The vast majority (96.9%) of patients fell from a height, while the rest fell from a height onto a sharp object. Most of them (90.9%) had experienced no shock symptoms. Upper limb injuries had the highest prevalence (37.8%), followed by lower limb injuries (22.7%), head injuries (19.7%) and skull fractures (13.6%). Invasive surgery, blood transfusions, admission to intensive care (ICU) and thoracostomy (chest tube) were required by 74%, 3%, 3% and 2% of patients, respectively.

**Conclusion:**

fall-related injuries may result in invasive surgery, chest drain insertion, or ICU admission, increasing the burden on the healthcare system.

## Introduction

Fall-related injuries in general, are an important global health concern, imposing an immense economic burden. Falls and fall-related injuries contribute to 10-15% of all emergency visits and 646,000 deaths worldwide every year [1]. Accidental falls impose significant morbidity, mortality and socioeconomic burden, rendering them as the second leading cause of hospitalization for all age groups around the world [2]. In the Kingdom of Saudi Arabia (KSA), approximately 28-35% people aged ≥65 years experience falls each year [3]. Fall-related injuries are the most common accidental injuries among children and adolescents in Riyadh [4]. Similarly, the prevalence of falls among hospitalized children is 9.9 per 1,000 in KSA [5]. The treatment of fall injuries is extremely expensive, further increasing the burden on the healthcare economy. In this context, the United States spends $50 billion and $754 million on non-fatal fall injuries and fatal fall injuries, respectively [6]. The incidence of falls and fall-related injuries increases with advancing age and degree of frailty [7]. Therefore, falls are common amongst the elderly, adding to their disability in terms of functional decline, anxiety, depression and reduced quality of life (QoL). Off these falls, >30% of falls require healthcare services and restricted activity [8]. Some of common causes of falls include functional decline, musculoskeletal diseases, neurological (i.e. cognitive, balance and sensory deficits) or psychosocial disorders and medications (alcohol, drugs) [9]. Fall injuries may involve any part of body, including extremities, head, neck, chest, abdomen, pelvis and spine.

In addition, cuts, bruises, sprains, strains, dislocations and fractures may be encountered [10]. Serious falls result in bone fractures, which lead to a prolonged hospital stay, disability, a decreased quality of life, multiple medications (polypharmacy) and increased cost [9]. Fall-related injuries typically result in minor trauma, such as bruises, abrasions, or lacerations. Therefore, most of the fall-related injuries are treated conservatively. However, serious injuries, such as intracranial injuries and fractures, can occur, requiring urgent treatment. Fractures of long bones, such as the femur and pelvis, may lead to significant post-fall morbidity and mortality [11]. In the elderly, the most common cause of serious injuries, such as traumatic brain injury and hip fractures, is from falls [12,13]. Therefore, the prevention of risk factors for falls in the elderly is of prime importance to reducing the disability and healthcare burden imposed by fall injuries. Most of the studies have been conducted related to the risks and falls of the elderly. The literature on the pattern of fall-related injuries and their management strategies covering all age groups is lacking. Falls are accidental injuries, which are preventable. Therefore, it is rightly said that prevention is better than treating. The present study aims at providing a pattern of fall injuries and their respective treatment, irrespective of age and gender. There is dearth of literature on the pattern of fall injuries, their severity and management strategies from the Saudi population. Therefore, this cross-sectional study was conducted to determine the pattern of fall injuries amongst the Saudi population and their treatment. It is a valuable addition to the literature about fall injuries and their management among the Saudi population.

## Methods

The aim of this study was to determine the characteristics of fall-related injuries and their management at a tertiary hospital in the Kingdom of Saudi Arabia (KSA). This was a cross-sectional study at the King Khalid Hospital and Prince Sultan Centre for Health Care (KKH&PSCHC) and other hospitals in Al Kharj for nine months from March 01, 2019 to November 30, 2019. The permission for the study was obtained from the ethical review board at KKH&PSCHC. A total of 264 patients were recruited for this study after they or the attendant signed informed consent forms.

In this study, fall was defined as “any unintentional fall from any height to the ground or lower level, not due to any organic cause” [14]. The inclusion criteria consisted of both male and female genders, patients of all ages, having provided informed consent, having resided in KSA and presenting to the King Khalid Hospital and Prince Sultan Centre for Health Care with at least one documented fall in their presenting complaint whether it was from a height or patients who fell while standing on the ground. Patients who had presented with a fall and other presenting complaints or pathologies were still included in the study. The exclusion criteria were minors who had not obtained consent from their legal guardians and participants who passed away in the emergency room and not transferred to the hospital ward. Participants who fell during their hospital stay were also excluded from this study. A total of 282 participants were screened for eligibility for the study and 264 were enrolled into the study. Eighteen participants did not meet all the inclusion and exclusion criteria. All 264 participants´ data were analysed for the study.

All participants who were recruited into the study were recruited in the emergency room after they had presented themselves. Their clinical documentation was followed through from triage all the way to patient discharge. The demographic details, aetiology of the fall, complications and prognosticating factors were retrieved from the patients´ electronic health records and tabulated in Microsoft Excel. Data was analysed using SPSS 22. Descriptive statistics were computed for study variables in terms of frequencies and percentages. Fisher exact test was applied to compare the study outcomes with other factors at significance level of 0.05. The interventions which were undertaken for the recruited patients were also documented; these included the insertion of a chest drain and invasive surgery. The disposition of the recruited patients was recorded as well; whether the patient was admitted to the general ward or the intensive care unit (ICU) was also tabulated.

## Results

**Figure 1:**
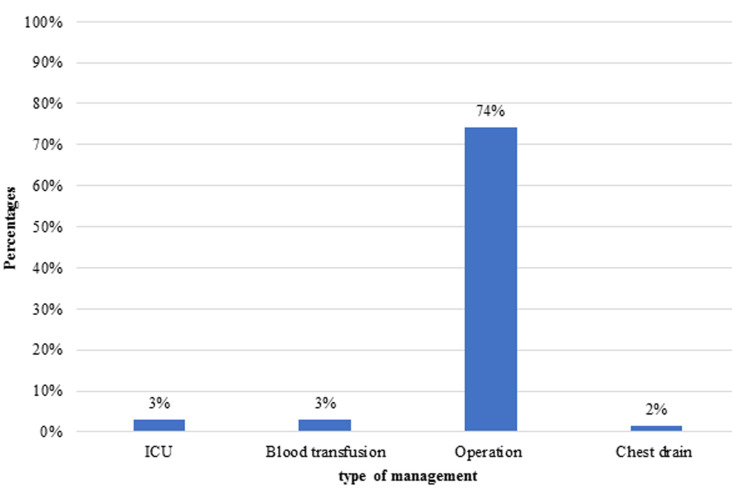
patient management

A total of 264 patients presented themselves to the King Khalid Hospital and Prince Sultan Centre for Health Services between March 01 and November 30, 2019 with injuries secondary to a fall. Most of these patients were children under the age of 10 (25.7%). This was followed by young adults between the ages of 21 and 30 (18.2%). The vast majority (77.2%) of patients were males. Other demographic details of the patients can be referenced in Table 1. Regarding the aetiology of falls (whether a fall was from body height or higher), the vast majority (96.9%) of patients fell from a height, while the remainder fell from a height onto a sharp object. Regarding the degree of shock experienced by patients, most (90.9%) showed no signs of shock, while 7.5% and 1.6% of them had first and second-degree shock, respectively. The region of injuries was documented for patients who were recruited into this study. Upper limb injuries had the highest prevalence at 37.8%, followed by lower limb injuries at 22.7%, head injuries at 19.7% and skull fractures at 13.6%. These details can be referenced in Table 2. In terms of the interventions undertaken for patients, as well as their disposition, 74% required invasive surgery for the management of their injuries, 3% required blood transfusions, 3% required ICU admissions and 2% required the insertion of a chest drain. Surgical intervention included, but were not limited to, open reduction and internal fixation (ORIF), closed reduction and internal fixation (CRIF), debridement, craniotomy and evacuation, laparotomy and nephrectomy. These are reflected in Figure 1.

**Table 1:** demographic details for study population (N=264)

Variable	N	%
**Nationality**		
Saudi	128	48.4%
Non-Saudi	136	51.6%
**Gender**		
Male	204	77.2%
Female	60	22.8%
**Age category**		
0-10	68	25.7%
11-20	36	13.6%
21-30	48	18.2%
31-40	36	13.7%
41-50	36	13.6%
51-60	28	10.6%
>60	12	4.6%

**Table 2:** region and injury type

Variable	N	%
Head injury (Yes)	52	19.7%
Skull fracture	36	13.6%
Brain damage	20	7.5%
Scalp laceration	8	3.0%
Chest injury	36	13.6%
Lung contusion	8	3.0%
Hemithorax	4	1.5%
Pleural effusion	8	3.0%
Rib fractures	28	10.6%
Abdomen injury	12	4.5%
Abdomen laceration	12	4.5%
Pelvis injury	28	10.6%
Pelvis fracture	24	9.0%
Pelvis laceration	4	1.5%
Spine injury	20	7.5%
Other injury	20	7.5%
Upper limb injury	100	37.8%
Humerus	20	7.5%
Elbow	4	1.5%
**Ulnar**		
Closed	28	10.6%
open	8	1.5%
Laceration wound	8	3.0%
Laceration wounds (multiple)	16	6.0%
Lower limb injury	60	22.7%
Femur	20	7.5%
Patella	8	3.0%
**Tibia**		
Closed	4	1.5%
Open	4	1.5%
Fibula (open)	4	1.5%
Ankle (closed)	16	6.0%

A two-way cross tabulation was performed for the type of injury as well as the insertion of a chest tube. The insertion of a chest drain associated strongly with chest injuries, abdominal injuries, pelvic injuries and other injuries (p<0.001). The cross-tabulation table can be found in Table 3. A two-way cross tabulation was performed for the type of injuries as well as invasive surgery, which was indicated for the management of these injuries. Invasive surgery strongly correlated with chest injuries, abdominal lacerations, rib fractures, spinal injuries and upper and lower limb injuries (p<0.001). The cross-tabulation table can be found in Table 4. A two-way cross tabulation was performed for the type of injury as well as ICU admission. ICU admissions strongly correlated with chest injuries, abdominal lacerations and pelvic lacerations (p<0.001). There were also strong associations observed between ICU admissions and brain damage, as well as other injuries (p=0.001). The cross-tabulation table can be found in Table 5.

**Table 3:** association between chest drain insertion and injury type

Tabulation of chest drain and chest injury					Tabulation of chest drain and abdomen laceration				
Chest drain	Chest injury			P-Value	Chest drain	Abdomen laceration			P-Value
	0	1	Total			0	1	Total	
0	228	32	260		0	252	8	260	
	100.00	88.89	98.48			100.00	66.67	98.48	
1	0	4	4		1	0	4	4	
	0.00	11.11	1.52			0.00	33.33	1.52	
Total	228	36	264	<0.001	Total	252	12	264	<0.001
	100.00	100.00	100.00			100.00	100.00	100.00	
**Tabulation of chest drain and pelvis injury**					**Tabulation of Chest drain an d Abdomen laceration**				
**Chest drain**	**Pelvis injury**			**P-Value**	**Chest drain**	**Abdomen laceration**			**P-value**
	0	1	Total			0	1	Total	
0	236	24	260		0	252	8	260	
	100.00	85.71	98.48			100.00	66.67	98.48	
1	0	4	4		1	0	4	4	
	0.00	14.29	1.52			0.00	33.33	1.52	
Total	236	28	264	<0.001	Total	252	12	264	<0.001
	100.00	100.00	100.00			100.00	100.00	100.00	
**Tabulation of chest drain and pelvis laceration**					**Tabulation of chest drain and other injury**				
**Chest drain**	**Pelvis laceration**			**P-Value**	**Chest drain**	**Other injury**			**P-Value**
	0	1	Total			0	1	Total	
0	260	0	260		0	244	16	260	
	100.00	0.00	98.48			100.00	80.00	98.48	
1	0	4	4		1	0	4	4	
	0.00	100.00	1.52			0.00	20.00	1.52	
Total	260	4	264	<0.001	Total	244	20	264	<0.001
	100.00	100.00	100.00			100.00	100.00	100.00	

**Table 4:** correlation between invasive surgery and injury type

Injury Types		Operation		Total	P-Value
		No	Yes		
Head injury	No	48(22.64%)	164(77.36%)	212(100%)	0.017*
	Yes	20(38.46%)	32(61.54%)	52(100%)	
Skull fracture	No	48(21.05%)	180(78.95%)	228(100%)	<0.001*
	Yes	20(55.56%)	16(44.44%)	36(100%)	
Abdomen laceration	No	68(26.98%)	184(73.02%)	252(100%)	0.026*
	Yes	0(0%)	12(100%)	12(100%)	
Spine fractures	No	52(21.31%)	192(78.69%)	244(100%)	<0.001*
	Yes	16(80%)	4(20%)	20(100%)	
Lower limb	No	64(31.37%)	140(68.63%)	204(100%)	<0.001*
	Yes	4(6.67%)	56(93.33%)	60(100%)	
Chest injury	No	44(19.3%)	184(80.7%)	228(100%)	<0.001*
	Yes	24(66.67%)	12(33.33%)	36(100%)	
Rib fracture	No	44(18.64%)	192(81.36%)	236(100%)	<0.001*
	Yes	24(85.71%)	4(14.29%)	28(100%)	
Spine injury	No	52(21.31%)	192(78.69%)	244(100%)	<0.001*
	Yes	16(80%)	4(20%)	20(100%)	
Upper limb	No	64(39.02%)	100(60.98%)	164(100%)	<0.001*
	Yes	4(4%)	96(96%)	100(100%)	
Total		68(25.76%)	196(74.24%)	264(100%)	

**Table 5:** association between ICU admission and injury type

Injury types		ICU		Total	P-Value
		No	Yes		
Head injury/brain damage	No	240(98.36%)	4(1.64%)	244(100%)	0.001*
	Yes	16(80%)	4(20%)	20(100%)	
Skull fracture	No	224(98.25%)	4(1.75%)	228(100%)	<0.014*
	Yes	32(88.89%)	4(11.11%)	36(100%)	
Abdomen laceration	No	248(98.41%)	4(1.59%)	252(100%)	<0.001*
	Yes	8(66.67%)	4(33.33%)	12(100%)	
Pelvis injury	No	232(98.31%)	4(1.69%)	236(100%)	0.005*
	Yes	24(85.71%)	4(14.29%)	28(100%)	
Pelvis laceration	No	256(98.46%)	4(1.54%)	260(100%)	<0.001*
	Yes	0(0%)	4(100%)	4(100%)	
Chest injury	No	228(100%)	0(0%)	228(100%)	<0.001*
	Yes	28(96.67%)	8(3.03%)	36(100%)	
Other injury	No	240(98.36%)	4(1.64%)	244(100%)	0.001*
	Yes	16(80%)	4(20%)	20(100%)	
Total		256(96.97%)	8(3.03%)	264(100%)	

## Discussion

This cross-sectional study determined the pattern of fall-related injuries and its management among people living in Saudi Arabia. Most victims of this type of injury were male children or adolescents. The most common fall-related injuries in KSA were injuries to the extremities (both upper and lower limbs), followed by injuries to head, chest and pelvis. Most fall-related injuries required urgent surgical procedures or operations, some requiring chest drain placement and ICU admission. The indication of chest drain placement was significantly high among those with chest, abdomen and pelvis injuries, as well as, among those with injuries labelled as “other injuries”. The requirement of surgical procedures had a significant correlation with chest (rib fracture), skull, chest, spine (fracture) and upper and lower limb injuries. Similarly, admission to the ICU was significantly associated with chest, brain, abdomen and pelvis injuries, as well as other injuries. It has been reported that falls are the principal cause of non-fatal injuries among children and adolescents, contributing to 50% fall-related disability-adjusted life years (DALYs) lost worldwide [15]. It shows that the present study correlates with the previous literature in terms of most common fall-related injuries occurring amongst children and adolescents, which imposes a significant burden on healthcare system. Fall-related injuries can occur at any age; however, children and the elderly are at higher risk of such injuries [16]. On the contrary, the present study reported the lowest number of fall-related injuries in people above 60 years of age.

It may be attributed to only 4.9% of the population above 60 years in KSA [3]. The present study is a good study that reports fall-related injuries irrespective of age and gender. Fall-related injuries may need surgical procedures, chest drain placement, blood transfusions and admission into the ICU. Patients suffering from fall-related injuries should be assessed thoroughly in the emergency room considering the rules of advanced trauma life support (ATLS). The patients with serious fall-related injuries require a multi-disciplinary approach and surgical intervention to address. Acute neurological, respiratory, or circulatory distress is usually a life-threatening condition, requiring immediate intervention via decompressive craniotomy, decompressive thoracostomy, haemostatic laparotomy, or pelvic fixation [17]. There are several traumatic conditions that require chest tube insertion (such as pneumothorax, tension pneumothorax, haemothorax and pleural effusion), increasing the risk of complications and morbidity [18,19]. Similarly, severe fall-related injuries (low GCS and high ISS) require admission to the ICU, especially for those with advanced age. In addition, the elderly suffer severe fall-related injuries with poor outcomes [20]. The strength of the present study is that it is a study from KSA, which has reported management strategies for the patients with fall-related injuries irrespective of age and gender. The limitations of the present study are that it is a single-centred study, short duration of study and that there is a lack of explanation regarding the detailed surgical procedures with their outcomes, especially in the form of mortality.

## Conclusion

Fall-related injuries may need urgent surgical procedures, chest drain insertions, or ICU admission, increasing the burden on the healthcare system. Although the first study showed the interventions and outcomes associated with fall-related injuries, further studies are warranted to look for specific surgical procedures required for specific fall-related injuries, as well as their outcome.

### What is known about this topic

- Falls and fall-related injuries contribute to 10-15% of all emergency visits;
- Approximately 646,000 die of fall-related injuries worldwide every year;
- Fall-related bone fractures lead to reduced quality of life and significant post-fall morbidity and mortality.

### What this study adds

- Majority of fall-related injuries (74%) required invasive surgery, increasing the hospital stay and the healthcare costs;
- Fall-related chest, abdomen and pelvis injuries often require chest drain placement. Similarly, rib or spine fracture, skull, chest and upper and lower limb injuries often require urgent surgical procedures and intensive care;
- As the present study reveals that most common sufferers of fall-related injuries were children and adults, the government of Saudi Arabia should construct improved policies and strategies to reduce such injuries at a young age.
